# Systemic inflammation attenuates the repair of damaged brains through reduced phagocytic activity of monocytes infiltrating the brain

**DOI:** 10.1186/s13041-024-01116-3

**Published:** 2024-07-29

**Authors:** Sushil Gaire, Jiawei An, Haijie Yang, Keon Ah Lee, Manisha Dumre, Eun Jeong Lee, Sang-Myun Park, Eun-Hye Joe

**Affiliations:** 1 Neuroscience Graduate Program, Department of Biomedical Sciences, Ajou University School of Medicine, Worldcup-ro 164, Suwon, Kyunggi-do 16499 South Korea; 2 Department of Pharmacology, Ajou University School of Medicine, Worldcup-ro 164, Suwon, Kyunggi-do 16499 South Korea; 3 Center for Convergence Research of Neurological Disorders, Ajou University School of Medicine, Worldcup-ro 164, Suwon, Kyunggi-do 16499 South Korea; 4 Department of Brain Science, Ajou University School of Medicine, Worldcup-ro 164, Suwon, Kyunggi-do 16499 South Korea

**Keywords:** Systemic inflammation, Brain injury, Monocytes, Phagocytosis, Repair

## Abstract

In this study, we examined how systemic inflammation affects repair of brain injury. To this end, we created a brain-injury model by stereotaxic injection of ATP, a damage-associated molecular pattern component, into the striatum of mice. Systemic inflammation was induced by intraperitoneal injection of lipopolysaccharide (LPS-ip). An analysis of magnetic resonance images showed that LPS-ip reduced the initial brain injury but slowed injury repair. An immunostaining analysis using the neuronal marker, NeuN, showed that LPS-ip delayed removal of dead/dying neurons, despite the fact that LPS-ip enhanced infiltration of monocytes, which serve to phagocytize dead cells/debris. Notably, infiltrating monocytes showed a widely scattered distribution. Bulk RNAseq analyses showed that LPS-ip decreased expression of genes associated with phagocytosis, with PCR and immunostaining of injured brains confirming reduced levels of Cd68 and Clec7a, markers of phagocytic activity, in monocytes. Collectively, these results suggest that systemic inflammation affects properties of blood monocytes as well as brain cells, resulting in delay in clearing damaged cells and activating repair processes.

**Supplementary Information:**

The online version contains supplementary material available at 10.1186/s13041-024-01116-3.

## Introduction

The peripheral immune system communicates with the central nervous system and affects brain function [1–4]. Systemic inflammation causes behavioral changes and cognitive dysfunction [5–7] by altering properties of brain cells [3, 8, 9]. Systemically administered lipopolysaccharide (LPS) activates not only blood cells but also astrocytes and microglia [3, 9, 10], resulting in increased blood-brain barrier (BBB) permeability [11] and vascular dysfunction owing to its effect on the endothelium [12]. Accordingly, chronic inflammatory diseases such as rheumatoid arthritis, atherosclerosis, diabetes and/or infection have been suggested as risk factors for central neurological diseases [4, 13, 14].

Communication between the peripheral immune system and brain cells may become more important in injured brains. In response to brain injury, blood monocytes enter the brain and participate in damage-repair processes [15–20]. Accordingly, depletion of monocytes impairs the repair of injured axons [21, 22], and formation of the BBB [23], thereby retarding recovery [16, 24]. Monocytes interact with neurons and astrocytes as part of the repair process, phagocytosing dying neurons and secreting extracellular vesicles containing neuronal fragments that guide elongation of neurites and astrocyte processes [15]. Monocytes activate astrocytes and abet their formation of a barrier around damage sites, thereby isolating themselves from intact brain regions in the later stages of repair [16, 25–27]. Astrocytes also play a role in the differentiation of monocytes into microglia – brain-resident macrophages [28]. Therefore, changes in the properties and/or function of monocytes can alter the repair of brain injury.

In this study, we examined how systemic inflammation affects the repair of injured brains. Systemic inflammation induced by intraperitoneal LPS injection (LPS-ip) increased monocyte infiltration into the injured brain. However, it decreased the phagocytic function of monocytes, resulting in delayed removal of damaged neurons and repair of injured brains.

## Materials and methods

**Figure:**
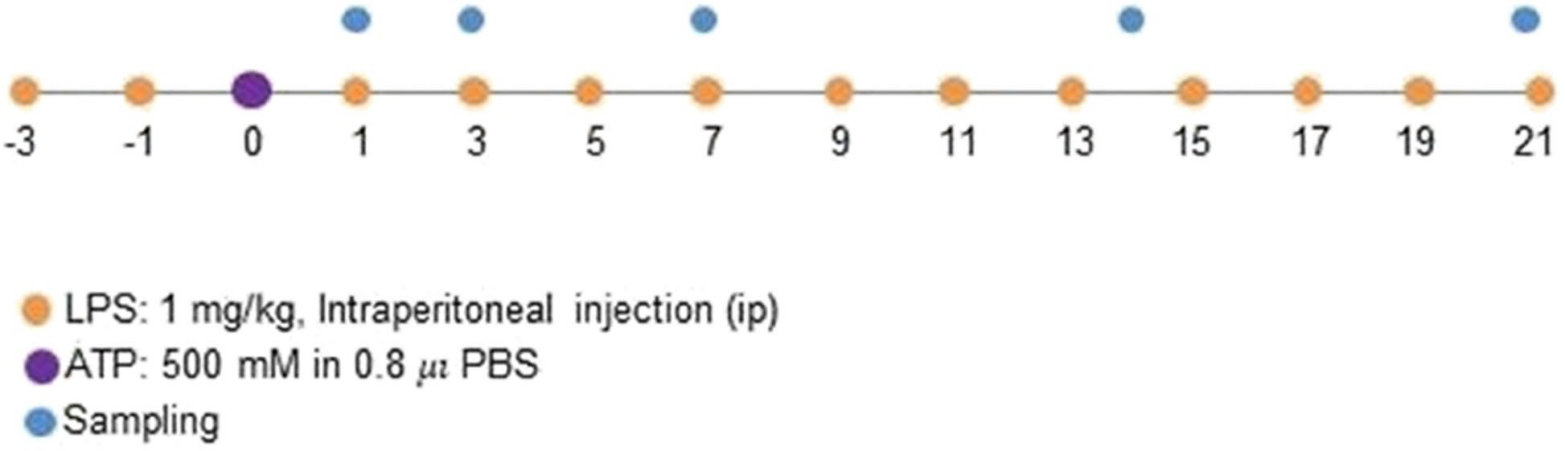
Research design. LPS (1 mg/kg, ip) was injected every other day beginning 3 d before injection of ATP (500 mM in 0.8 μl PBS) into the striatum, continuing until the animal was sacrificed

### Animals and LPS injection

C57BL/6N, male mice of 8–10 weeks old were used in this study. Mice were kept under a 12-hr light/dark cycle with free access to food and water. All experiments were conducted in accordance with approved animal protocols and guidelines established by the Ajou University School of Medicine Ethics Review Committee for animal experiments.

A group of animals were challenged with LPS (1mg/kg) (L7770, sigma, from salmonella enterica serotype enteritidis) intraperitoneally on an alternate day starting 3d before brain injury and continuing till the day of sacrifice.

### Stereotaxic injection

Mice were anesthetized with intraperitoneal injections of tribromoethanol (250 mg/kg; Sigma, St. Louis, MO) and secured in a stereotaxic apparatus (David Kopf Instruments, Tujunga, CA). Brain injury was induced by unilateral administration of ATP (500 mM, 0.8 μl; Sigma), a DAMPs component, into the striatum (AP, +1.0 mm; ML, −2.0 mm; DV, −3.2 mm from bregma) based on stereotaxic coordinates from The Atlas of the Mouse Brain (Paxinos and Franklin, 2nd edition), as detailed in previous work [29]. ATP was prepared by dissolving it in sterile phosphate-buffered saline (PBS), and a total of 0.8 μl of ATP solution was infused at a rate of 0.2 μl/min using a Hamilton syringe equipped with a 30-gauge needle attached to a syringe pump (KD Scientific, New Hope, PA). The total injection time, encompassing 4 minutes of ATP infusion and an additional waiting period of 6 minutes before withdrawal, aimed to prevent leakage or outflow.

### Magnetic resonance imaging (MRI)

Volumetric changes of injured striatum was tracked for 15 d using a 9.4 T MR scanner (BioSpec 94/30 US/R; Bruker, Billerica, MA, USA) at Sungkyunkwan University (Suwon, Korea). Mice were briefly anesthetized with 1.5% isoflurane. Respiration and body temperature were continuously assessed using an MR-compatible small animal monitoring and gating system (Model 1025; SA Instruments, Inc., Stony Brook, NY, USA). T2-weighted 2D Turbo rapid imaging with a refocused echo (RARE) sequence was performed using the following parameters: repetition time (TR) = 9000 ms; echo time (TE) = 33 ms; resolution, 78 μm×78 μm× 250 μm; thickness, 250 μm; RARE factor, 8; average, 2; scan time, 10 min. MR images were analyzed using Mimics software (Materialise, Leuven, Belgium).

### Tissue preparation

Mice were anesthetized and transcardial perfusion was done initially with PBS containing 0.5% sodium nitrate and heparin (10 U/ml), followed by perfusion with 4% paraformaldehyde (PFA) in 0.1 M phosphate buffer (PB; pH 7.2). Subsequently, the brains were stored at 4 °C in 4% paraformaldehyde in 0.1 M PB for 1 day and then transferred to 30% sucrose in PBS until complete sinking occurred. Using a cryostat (Leica, Wetzlar, Germany), six separate series of 35-μm-thick coronal sections were obtained and stored in an antifreeze stock solution (PB containing 30% glycerol and 30% ethylene glycol, pH 7.2) at 4 °C until utilized for immunostaining.

For protein and RNA sampling, brains were collected after transcardial perfusion with PBS. Brain slice (2 mm thickness) of the needle injection site was prepared using an Alto mouse brain slicer matrix (Roboz Surgical Instruments, Gaithersburg, MD, USA). Tissue blocks (2×2×2 mm^3^) were obtained and stored at − 70 °C until use.

### RNA sequencing and data analysis

Total cellular RNA was extracted using the RNeasy kit (Qiagen, Valencia, CA) following the manufacturer's instructions. Illumina-compatible libraries were generated with the TruSeq RNA library preparation kit (Illumina, CA, USA), as per the manufacturer's guidelines.

Macrogen (Seoul, Korea) conducted whole transcriptome sequencing. To summarize, mRNA was purified through polyA selection from total RNA, chemically fragmented, and converted into single-stranded cDNA using random hexamer priming. RNA libraries were then constructed through PCR amplification, and quantification was performed using quantitative PCR (qPCR) with an Agilent 2100 Bioanalyzer, following the qPCR Quantification Protocol Guide (Agilent Technologies Inc., San Diego, CA). The cDNA library underwent sequencing on the HiSeq2000 platform (Illumina), and Macrogen conducted the sequence analyses. Quality verification of the sequences was done using FastQC v0.10.0. Raw data were log2 transformed and subjected to analysis using R Studio software.

### Immunostaining

For immunofluorescence staining, sections were washed with PBS containing 0.2% Triton X-100 (PBST), treated with 1% bovine serum albumin (BSA), and incubated first with combinations of primary antibodies overnight at 4 °C. The sections were rinsed three times with PBST and incubated with biotinylated secondary antibodies. Nuclei were visualized by counterstaining with 4’, 6-diamidino-2-phenylindole (DAPI; Sigma). Sections were embedded in Fluoroshield Mounting Medium (Abcam, Cambridge, Great Britain). Details of antibodies can be found in Table 1.

**Table 1:** Antibodies used in this study

Antibodies	Dilution factors for	
	Western blotting	Immunostaining
Rabbit anti-CD45 (abcam, ab10558)	1:1000	1:1000
Rat anti-CD45 (mybiosource, mbs520149)	N/A	1:1000
Rabbit anti-Iba-1 (Wako, 019-19741)	1:1000	N/A
Rabbit anti-GFAP	N/A	1:1000
Mouse anti-GFAP (Sigma, 3893)	1:1000	N/A
Rabbit anti-NEUN (abcam, ab177487)	N/A	1:1000
Rabbit anti-CD68 (abcam, ab125212)	N/A	1:1000
Rabbit anti-GAPDH (cell signaling, 5174s)	1:5000	N/A
Rat anti-Dectin-1 (mabg-mdect)	N/A	1:200
DAPI (Sigma-Aldrich, D9542)	N/A	1:1000
Dylight 488 tomato lectin (Vectors lab, DL 11741)	N/A	1:100
Donkey anti-rabbit IgG (H+L), Alexa Fluor 488 (Invitrogen A21206)	N/A	1:500
Donkey anti-rabbit IgG (H+L), Alexa Fluor 555 (Invitrogen A31572)	N/A	1:500
Donkey anti-rabbit IgG (H+L), Alexa Fluor 647 (Invitrogen A31573)	N/A	1:500
Donkey anti-rat IgG (H+L), Alexa Fluor 488 (Invitrogen A21208)	N/A	1:500
Donkey anti-rat IgG (H+L), Alexa Fluor 594 (Invitrogen A21209)	N/A	1:500

“Not Applicable” (NA) signifies that the respective antibody was not used in the study, either for Western blotting or Immunostaining

Fluoro-Jade C (FJC) staining was performed following the immunofluorescence staining procedure. For FJC staining sections underwent treatment with a 0.06% potassium permanganate solution for 10 minutes, were rinsed with water, and then exposed to 0.0001% Fluoro-Jade C (Merck, Darmstadt, Germany) in 0.1% acetic acid for 10 minutes, following the manufacturer's instructions. Subsequently, images were captured using an LSM 800 confocal microscope (Carl Zeiss, Oberkochen, Germany).

### Isolation of peritoneal macrophages and peripheral blood mononuclear cells (PBMCs)

Peritoneal macrophages were acquired from WT mice by rinsing the peritoneal cavity with sterile PBS. Following this, the collected cells underwent centrifugation to isolate peritoneal macrophages. Simultaneously, peripheral blood mononuclear cells (PBMCs) were obtained via cardiac puncture and isolated using density gradient centrifugation with a Ficoll gradient. To eliminate red blood cells, the isolated PBMCs were washed with PBS and RBC lysis buffer. The resulting pellet was utilized for PCR analysis.

### Total protein extraction and Western blotting

Total protein extraction was performed on ice using RIPA buffer (10mM PB pH 7.2, 150mM NaCl, 1% NP-40, 0.5% sodium deoxycholate) supplemented with a protease/phosphatase inhibitor cocktail (GenDEPOT, Barker, TX, USA). The proteins were denatured by incubating for 5 minutes at 95 °C in sample buffer (6.25mM Tris pH 6.8, 12.5% glycerol, 2.5% SDS, 0.025% bromophenol blue, and 5% β-mercaptoethanol), separated through sodium dodecyl sulfate-polyacrylamide gel electrophoresis (SDS-PAGE), and subsequently transferred to a nitrocellulose membrane (GE Healthcare, Pittsburgh, PA, USA). Following blocking with 5% skim milk (Seoul Dairy Coop, Seoul, Korea), the nitrocellulose membrane underwent sequential incubation with primary antibodies, peroxidase-conjugated secondary antibodies (Koma Biotech, Seoul, Korea), and enhanced chemiluminescence reagents (Daeil Lab Services, Seoul, Korea). Glyceraldehyde 3-phosphate dehydrogenase (GAPDH) served as the loading control. Band intensities were quantified using Image J.

### Quantitative real-time polymerase chain reaction (QPCR) and Reverse Transcriptase PCR (RT-PCR)

Total RNA was isolated from injured mouse brains (striatum), peritoneal macrophages and peripheral blood mononuclear cells (PBMCs), using easy-BLUE reagent (iNtRON Biotechnology, Seongnamsi, Korea). cDNA synthesis was performed using a cDNA synthesis kit (iNtRON Biotechnology) following the manufacturer’s guidelines, starting with 1 μg of total RNA. The cDNA, along with forward and reverse primers (1 μM), was mixed with 2X Kapa SYBR Fast Master Mix (Kapa Biosystems, Boston, MA, USA). Quantitative real-time reverse transcription-polymerase chain reaction (RT-qPCR) was conducted using a Step-One Real Time PCR System. Threshold cycle values were calculated for each gene and normalized to GAPDH, serving as an internal reference. For RT-PCR, the Reverse Transcription Master Premix (ElpisBio, Daejeon, Korea) was employed according to the manufacturers’ instructions. Validation of RT-PCR products was achieved through electrophoresis on 1.5% agarose gels stained with GelRed (Biotium, Hayward, CA, USA). GAPDH served as the reference, and band intensities were analyzed using Quantity One 1-D analysis software, v 4.6.5 (Bio-Rad Laboratories, Inc., Hercules, CA, USA). Primer sequences are detailed in Table 2.

**Table 2:** Primer sequences for real-time qPCR analysis

mRNA	Oligonucleotide Sequence (5’-3’)	PCR type
*Il1b*	F: TCCTGTGTAATGAAAGACGGC R: ACTCCACTTTGCTCTTGACTT	Conventional
*Tnfa*	F: CTTCTGTCTACTGAACTTCGG R: CAGGCTTGTCACTCGAATTTT	Conventional
*Cd68*	F: GACCTACATCAGAGCCCG R: CGCCATGAATGTCCACTG	qPCR
*Clec7a*	F: AGACTCATCTGCTATGCTGCC R: AACGGGAGAGCTAAAGGCAC	Conventional
*Ccr2*	F: GGCTTATCCAAGCATGGTGATTTAG R: ACCACTTGCATGCACACATGA	qPCR
*Ccl5*	F: GGAGTATTTCTACACCAGCAGCAAG R: GGCTAGGACTAGAGCAAGCAATGAC	qPCR
*Gapdh*	F: CCACCCCAGCAAGGAGACT R: GAAATTGTGAGGGAGATGCT	qPCR & Conventional

### Statistical analysis

Statistical analyses were carried out using GraphPad Prism software (Prism; San Diego, CA). To compare two groups, "unpaired t-test" was utilized. Multiple group comparisons were conducted through two-way analysis of variance. The specific statistical tests, along with corresponding p values, are also delineated in the figure legends. The difference was considered significant when *p* < 0.05. All reported values represent means ± standard errors of the means (SEMs) obtained from at least of three independent experiments.

## Results

### Transcriptome analysis of injured brains of mice with and without LPS-ip

To examine how systemic inflammation affects brain injury and repair, we induced systemic inflammation by ip injection of LPS (1 mg/kg) every other day (Fig. Illustration of research design). Brain damage was induced by stereotaxic striatal injection of ATP, a component of damage-associated molecular pattern (DAMP) that acutely induces damage suitable for quantitative analysis [29–31]. Striatum is associated with several brain diseases including Parkinson disease, Huntington disease, and addiction [32, 33]. The induction of systemic inflammation was confirmed by increased mRNA levels of interleukin *(Il)-1β* and tumor necrosis factor (Tnf)-α in peritoneal macrophages and peripheral blood mononuclear cells (PBMCs). Specifically, *Il1-β* mRNA levels increased in both types of cells from 1 d to 15 d, whereas *Tnfα* levels strongly and transiently increased at 1 d in peritoneal macrophages and showed a weak but sustained increase in PBMCs (SFig. 1). LPS-ip also increased protein levels of Iba-1 (a marker of microglia) and GFAP (a marker of astrocytes) (SFig. 1), with Western blot analyses showing an increase in Iba-1 within 1 d that was maintained for at least 21 d and GFAP slightly increasing at 7 and 14 d before decreasing to basal levels at 21 d.

**Fig. 1:**
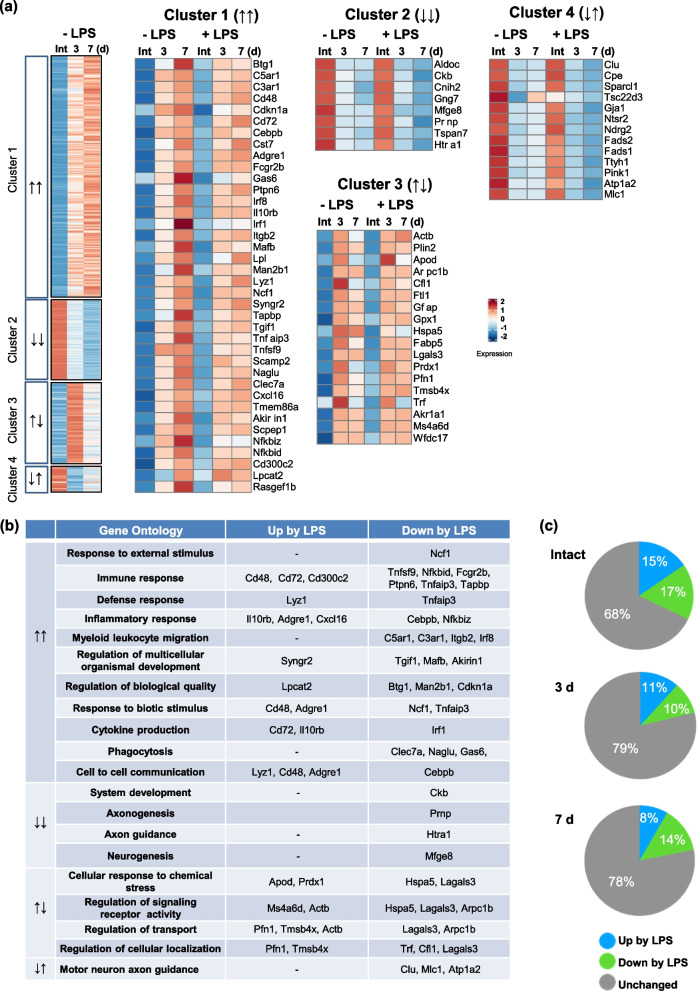
Transcriptome analysis of injured brains. **a** Heatmap displaying gene expression in the control group at the indicated time points obtained from bulk RNA sequencing (RNA-Seq) analysis of the striatum, categorized into four groups by K-means clustering (scale = Z scores of TPM values and genes with TPM value >10 in at least one time point were selected). **b** Gene Ontology (GO) terms assigned to each cluster, together with gene names based on their upregulation and downregulation in response to LPS-ip. **c** Pie chart showing the percentage of genes regulated by LPS-ip at each comparison (*p* < 0.05)

We first analyzed transcriptomes of injured brains with and without LPS-ip using a bulk RNA sequencing (RNA-Seq) analysis at 3 and 7 d post ATP-injection, a time when the repair of injury is actively ongoing [15, 18, 21]. Based on the patterns of gene expression across time points without LPS-ip, we obtained four clusters of genes by applying the K-means clustering technique using R studio (Fig. 1a): cluster 1, continuously increased at 3 and 7 d (↑↑); cluster 2, continuously decreased (↓↓); cluster 3, increased at 3 d and then decreased at 7 d (↑↓); and cluster 4, decreased at 3 d and then increased at 7 d (↓↑). Genes in cluster 1 (↑↑) – *C5ar1*, *Fcgr2b*, *Clec7a*, *Irf8*, *Itgb2*, *Gas6*, and *Irf1* – were associated with the immune system, inflammation, myeloid leukocyte migration, cytokine production, and phagocytosis (Fig. 1a, b). Genes in clusters 2 (↓↓) and 4 (↓↑) – *Atp1a2*, *Prnp*, *Htra1*, *Mlc1*, *Pink1*, *Ckb* and *Mfge*8 – were mostly those associated with repair and regeneration processes, including axonogenesis, neurogenesis, and axon guidance (Fig. 1a, b). Genes in cluster 3 (↑↓) – *Hspa5*, *Actb*, *Tmsb4x* and *Pfn1* – were related to signaling and transport (Fig. 1a, b). The proportions of genes that showed decreased or increased expression in response to LPS-ip were similar in intact (upregulated, 15%; downregulated, 17%) and 3-d post-injury (upregulated, 11%; downregulated 10%) groups (Fig. 1c). At 7 d, the proportion of genes downregulated by LPS-ip (14%) was greater than that of upregulated genes (8%) (Fig. 1c).

### LPS-ip reduces initial brain damage but delays removal of dead neurons and repair of injured brains

**Fig. 2:**
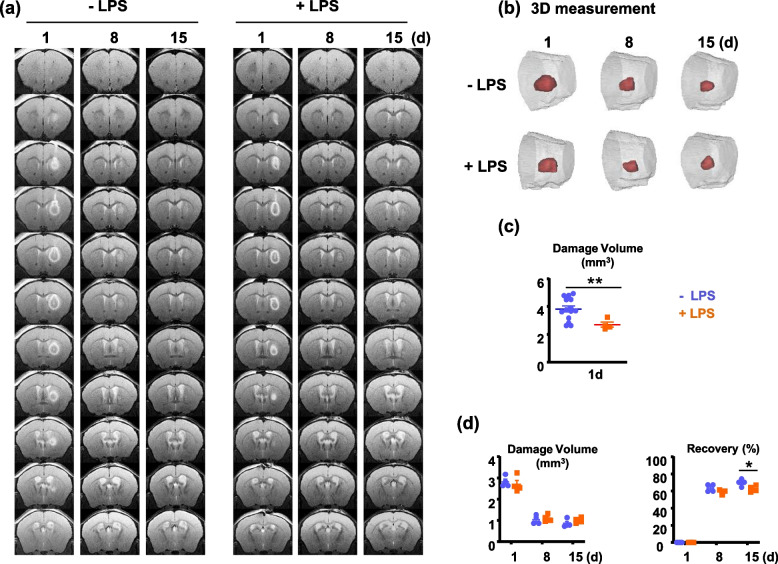
Time course of changes in injury volume following LPS-ip. **a** Damage was induced by stereotaxic injection of ATP (500 mM in 0.8 μl PBS) and analyzed with 9.4T MR. Serial coronal images were acquired every 250 μm. **b, c, d** 3D structures of damage regions reconstructed based on MR images using Mimics software (b) and calculation of initial damage volumes (c) and recovery (d). The recovery rates of injuries with size between 2.5 - 3.5 mm^3^ were measured. Values are means ± SEMs of at least 4 mice (*p < 0.05, **p < 0.01, for -LPS vs. +LPS; unpaired t-test)

Next, we analyzed changes in brain damage by magnetic resonance imaging (MRI) using 9.4T MR. Damage volumes were calculated based on 3D structures reconstructed from MR images (Fig. 2). The initial damage was somewhat smaller in LPS-ip brains than control brains (Fig. 2b, c). Next, we compared damage recovery rates in control brains and found that these recovery rates depended on initial damage area: rates were high for large damage areas and low for small damage areas (SFig. 2). To further assess damage, we used a normalized approach, comparing recovery rates of similar-sized injuries in each group; for this, we chose volume of ~3 mm^3^ (average brain damage size in LPS-ip animals) (Fig. 2d). This analysis showed that damage volume was slightly attenuated in LPS-ip brains compared with control brains (Fig. 2d).

**Fig. 3:**
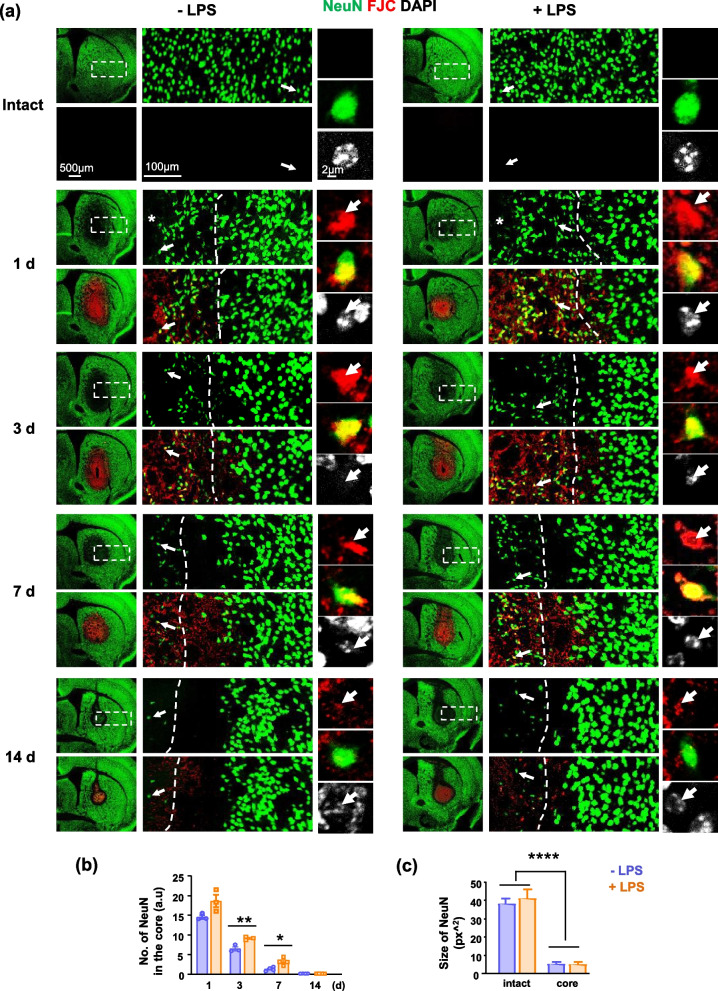
Effect of LPS-ip on neuronal changes in the injured brain. **a** Brain sections from control and LPS-ip mice with ATP-induced brain damage, obtained at the indicated times, were stained with NeuN (neuronal marker) and FJC (degenerating neuron marker). Dotted lines separate small size NeuN(+) cells in the core from intact region, and arrows indicate the FJC(+) NeuN cells. **b, c** The number of NeuN in the injury core (b), and the size (c) of NeuN in the core and intact regions were measured using Image J. Values are means ± SEMs of 3 mice (*p < 0.05, **p < 0.01, ****p < 0.0001, for -LPS vs. +LPS; unpaired t-test)

We further analyzed changes in neurons in injured brains by immunostaining for the neuronal marker, NeuN. In both LPS-ip and control brains, NeuN(+) cells in the damage core (area 1: the left side of the dotted lines) were small compared with those in intact regions (area 2; the right side of the dotted lines) (Fig. 3a, c). These small NeuN(+) cells (arrows in Fig. 3a) were positive for staining with Fluoro-Jade C (FJC), a marker of degenerating neurons [34], and their nuclei stained with 4’,6-diamidino-2-phenylindole (DAPI) were shrunken and condensed, a morphology characteristic of dead/damaged cells [35]. The number of small NeuN(+) cells in the core decreased between 1 and 14 d post injury. Notably, however, there were more small cells in LPS-ip brains than in control brains at all time points (Fig. 3a, b). These results indicate that dead/dying cells remained longer in the injury core of LPS-ip brains than in control brains, despite the fact that brain damage was smaller in LPS-ip brains.

### LPS-ip enhances infiltration of monocytes but attenuates their phagocytic activity

**Fig. 4:**
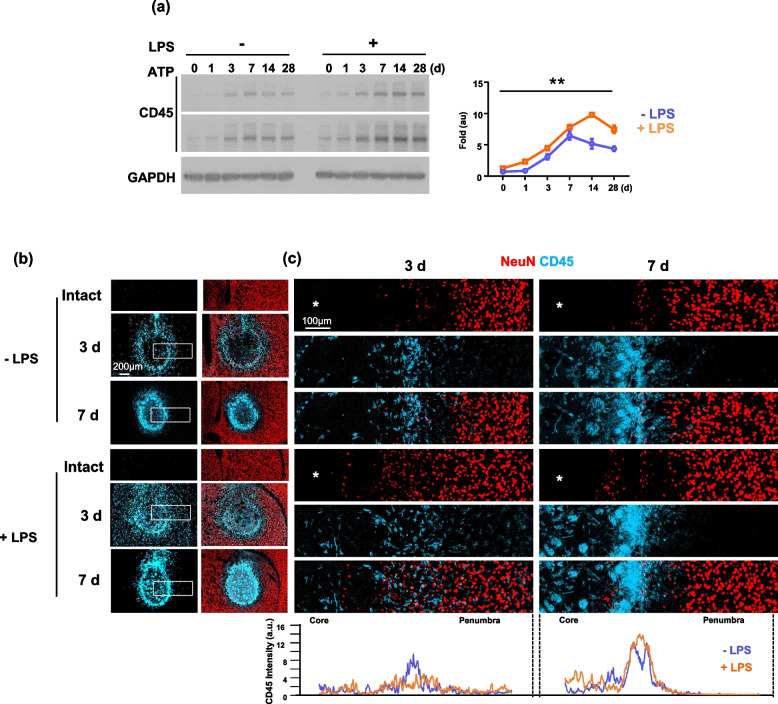
Effect of LPS on monocyte infiltration in the injured brain. Brain lysates (**a**) or sections (**b**, **c**) were obtained at the indicated times after ATP injection. **a** Monocyte infiltration was analyzed by Western blot analysis using antibodies for CD45; band intensities were analyzed using Image J. Values are means ± SEMs of 3 mice (**p < 0.01, for -LPS vs. +LPS; two-way ANOVA). **b, c** Brain sections stained with antibodies for NeuN (red) and CD45 (blue). Magnified images of areas in (**b**) are shown in (**c**). The distribution of monocytes in (c) was analyzed using Image J

It has been reported that monocytes that infiltrate into the injured brain remove dead cells and debris [15, 18, 36–38]. Accordingly, we hypothesized that delayed removal of damaged cells in LPS-ip brains could be related to monocyte infiltration and/or phagocytic activity. In both control and LPS-ip mouse brain lysates, levels of CD45, a monocyte-specific marker, were detectable within 1 d after injury, reached a peak at 7-14 d, and then declined (Fig. 4a). Unexpectedly, CD45 levels were slightly higher in LPS-ip brains (Fig. 4a and SFig. 3). Immunostaining also showed greater infiltration of monocytes in injured LPS-ip brains than in control brains (Fig. 4b, c). Monocytes were distributed around the damage core at 3 d and became more densely located at 7 d (Fig. 4b, c). Interestingly, in LPS-ip brains, monocytes were widely scattered with a sparser distribution in the area where dying neurons remained, particularly at 3 d (Fig. 4c).

**Fig. 5:**
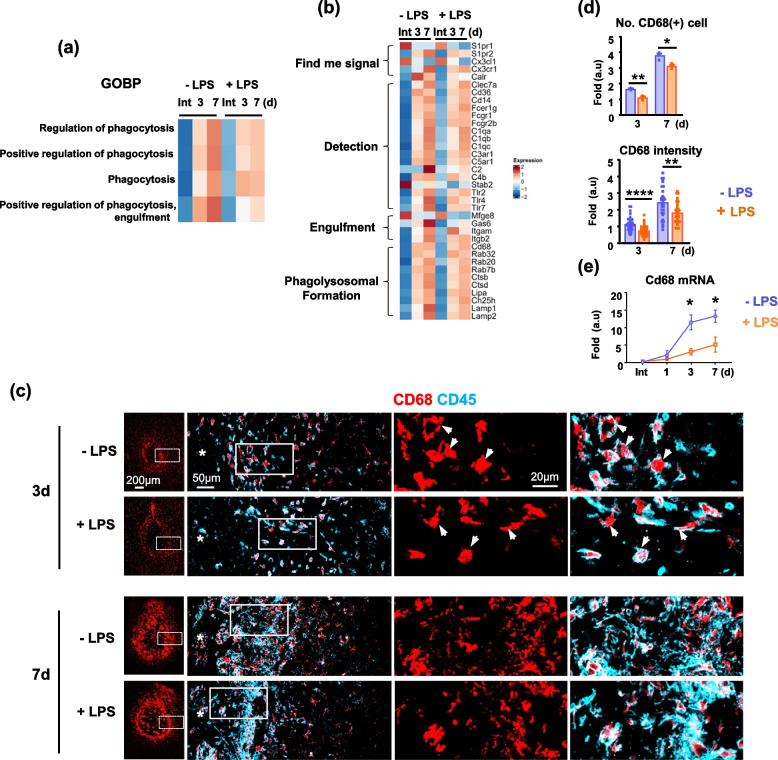
LPS decreases CD68 expression in the injured brain. **a** A transcriptomic analysis of the top four GO terms under phagocytosis identified through gene enrichment examination and their gene set variation analysis (GSVA) at the indicated time points. **b** Heatmap illustrating differences in the expression of genes related to different stages of phagocytosis between the two groups at the indicated time points (scale = Z scores of TPM values). **c** Brain sections stained with antibodies for CD68 and CD45. Arrows indicate CD45(+)/CD68(+) monocytes. **d** Numbers of CD68(+) cells and intensity of CD68 staining, analyzed with Image J and Zen software. Values are means ± SEMs of 3 mice. **e**Total RNA was isolated at the indicated times after injury as described in Method, and *CD68* mRNA levels were measured by quantitative PCR (qPCR). Values are means ± SEMs of 3 mice (*p < 0.05, **p < 0.01, ****p < 0.0001, for -LPS vs. +LPS; unpaired t-test)

A transcriptome analysis revealed increase in expression of the phagocytosis-related genes in ATP-injured brains of control and LPS-ip mice at 3 and 7 d (Fig. 5a). It is noticeable that LPS-ip increased expression of phagocytosis-related genes less at all four stages of phagocytosis, find-me-signal (for example, *S1pr2)*, detection (*Clec7a)*, engulfment (*Gas6)*, and phagolysosomal formation (*CD68* and *Lamp2*) at 7 d post injury (Fig. 5b). Immunostaining showed that CD68, a marker of lysosomal activity [18, 36, 39], was detectable in CD45(+) monocytes at 3 and 7 d post injury (Fig. 5c). Consistent with the transcriptomic analysis, both the number of CD45(+) monocytes that expressed CD68 and the intensity of CD68 immunostaining in monocytes were higher in the injured brains of control mice than those of LPS-ip mice (Fig. 5c, d). CD68 mRNA levels were also higher in injured brains of control mice than those of LPS-ip mice (Fig. 5e).

**Fig. 6:**
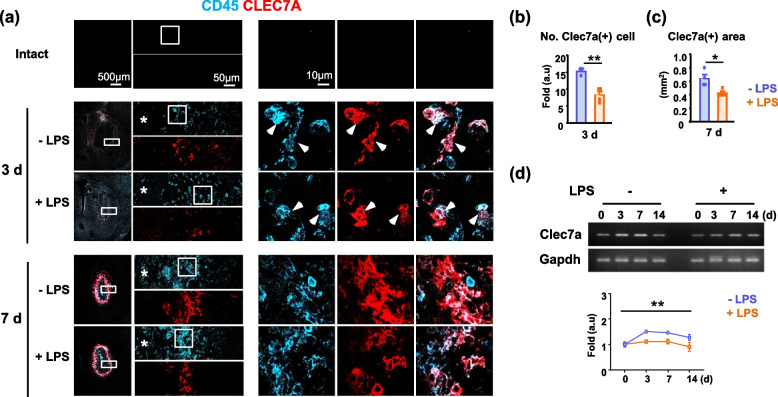
LPS decreases Clec7a expression in the injured brain. **a** Brain sections stained with antibodies for CD45 and Clec7a. **b, c** Analysis of Clec7a(+) number and area with Image J. Arrows indicate CD45(+)/Clec7a(+) monocytes. Values are means ± SEMs of 3 mice (*p < 0.05, **p < 0.01, for -LPS vs. +LPS; unpaired t-test). **d** Total RNA was isolated at the indicated times after injury as described in Method. *Clec7a* mRNA levels were measured using conventional RT-PCR, and the band intensities were measured. Values are means ± SEMs of 3 mice (**p < 0.01, for -LPS vs. +LPS; two-way ANOVA)

Immunostaining of Clec7a (dectin-1), a pattern-recognition receptor that activates phagocytosis [40, 41], showed a reduction in intensity in monocytes of LPS-ip brains compared with control brains at 3 and 7 d post-injury (Fig. 6a–c). Consistent with this, Clec7a mRNA levels were also decreased in LPS-ip brains (Fig. 6d). These results suggest that removal of dying/dead cells by monocytes in injured LPS-ip brains is attenuated owing to the dispersed distribution and reduced phagocytic activity of infiltrated monocytes.

## Discussion

Systemic inflammation induced by LPS-ip hindered the timely elimination of damaged neurons. Inadequate repair may accumulate damage, leading to a progressive loss of brain function and neurodegeneration. Monocytes that infiltrate into the injured brain play a role in repairing the injury. Therefore, the altered function of monocytes may alter the repair process of brain injury. The results of this study provide clues about how various inflammatory diseases, including diabetes and arthritis, may be risk factors for diverse brain diseases.

Despite the delay in eliminating dying cells, initial injury was reduced in LPS-ip brains. It has been reported that LPS increases expression of inflammatory mediators through activation of microglia and astrocytes, thereby inhibiting the inflammatory response to subsequent brain injury [42, 43]. Thus, LPS-ip may act as a preconditioning effect for neuroprotection.

Systemic inflammation enhanced the infiltration of monocytes, but delayed the elimination of damaged neurons, indicating that monocyte recruitment factor is strengthened but the elimination factor is reduced. We found that mRNA levels of chemokines/chemokine receptors such as *Ccl5, Ccl12, Ccl3, Ccl27a*, and *Ccr2* were higher in injured LPS-ip brains than in injured control brains (SFig. 4). However, the morphological features of blood vessels (length and number of branches) through which monocytes enter the brain were similar in before and after the injury, and similarly decreased in both groups by the injury (SFig. 5).

The spatial distribution of phagocytes is an important determinant of the efficiency of phagocytosis [44, 45]. In injured LPS-ip brains, monocytes localized to the penumbra region were more widely distributed with a lower density than those in WT injured brains, factors that may have contributed to the delayed removal of dying neurons. In addition, the markers of phagocytic activity, CD68 and CLEC7A, were overlapped with CD45(+)-monocytes. Microglia phagocytose relatively small structure such as synapses (synapse pruning) [46, 47].

To investigate factors that regulate monocyte distribution, we examined astrocytes, known for forming barriers that limit immune cell access to the injury core [48, 49]. Unexpectedly, however, in injured LPS-ip brains, GFAP-stained astrocytes displayed a more activated morphology and formed tighter barriers at 3 d post injury, as evidenced by an increase in total GFAP(+) area, cell body area, and process numbers (SFig. 6). Therefore, factors other than astrocyte barrier formation may act for the dispersed distribution of monocytes in injured LPS-ip brains.

Monocytes displayed defects in phagocytic function, which might delay the removal of damaged cells and debris. Because MR images and transcriptome analyses showed that LPS-ip reduced the initial damage, we ruled out the possibility that the increased presence of unhealthy cells in the LPS-ip brain was attributable to more severe damage. As expected, transcriptome analyses showed that LPS-ip reduced the expression of genes associated with various stages of phagocytosis, including “find me/eat me” (e.g., *S1pr1*, *Cx3cr1*, *Calr*), detection (e.g., *Clec7a*, C*d36*, *Fcgr*, *C1q*), engulfment (e.g., *Mfge8, Gas6*), and degradation (e.g., *Cd68*, *Ctsb*, *Lamp2*) genes. Thus, LPS-ip may affect the efficiency of phagocytosis from early stages (find me/eat me and detection) to late stages (engulfment and degradation). Consistent with this, in injured LPS-ip brains, CD68 and Clec7a expression were reduced in monocytes, showing that monocytes did not effectively clear damaged neurons. Previous studies support these findings, demonstrating that the phagocytic ability of monocytes can be decreased by an inflammatory milieu [50–52]. Therefore, systemic inflammation attenuate removal of dead cells and debris, which delays repair of injured brains and/or accumulates injury.

### Supplementary Information

Supplementary Material 1.

## Data Availability

Kindly reach out to the author regarding requests for data and materials.

## Abbreviations

LPS-ip: Intraperitoneal injection of lipopolysaccharide
FJC: Fluoro-jade C
PBMCs: Peripheral blood mononuclear cells
NeuN: Neuronal nuclear antigen
ATP: Adenosine triphosphate
MR: Magnetic resonance
GFAP: Glial fibrillary acidic protein
Gapdh: Glyceraldehyde 3-phosphate dehydrogenase
CD45: cluster of differentiation 45
Cd68: Cluster of differentiation 68
Clec7a: C-type lectin domain containing 7A
Iba-1: Ionized calcium binding adaptor molecule 1
DAPI: 4’,6-diamidino-2-phenylindole
Il-1β: Interleukin 1 beta
Tnf-α: Tumor necrosis factor-alpha
PBS: Phosphate-buffered saline
RT-PCR: Reverse Transcriptase PCR
qPCR: Quantitative real-time polymerase chain reaction
Ccr2: C-C chemokine receptor type 2
Ccl5: C-C motif chemokine ligand 5
