# Supplementary material for: Systemic inflammation attenuates the repair of damaged brains through reduced phagocytic activity of monocytes infiltrating the brain
Source: Mol Brain. 2024 Jul 29;17:47. doi: 10.1186/s13041-024-01116-3 (PMC11288066; doi:10.1186/s13041-024-01116-3)
Supplement: Supplementary file 1 — Supplementary Material 1. [file 13041_2024_1116_MOESM1_ESM.pptx]

## Slide 1
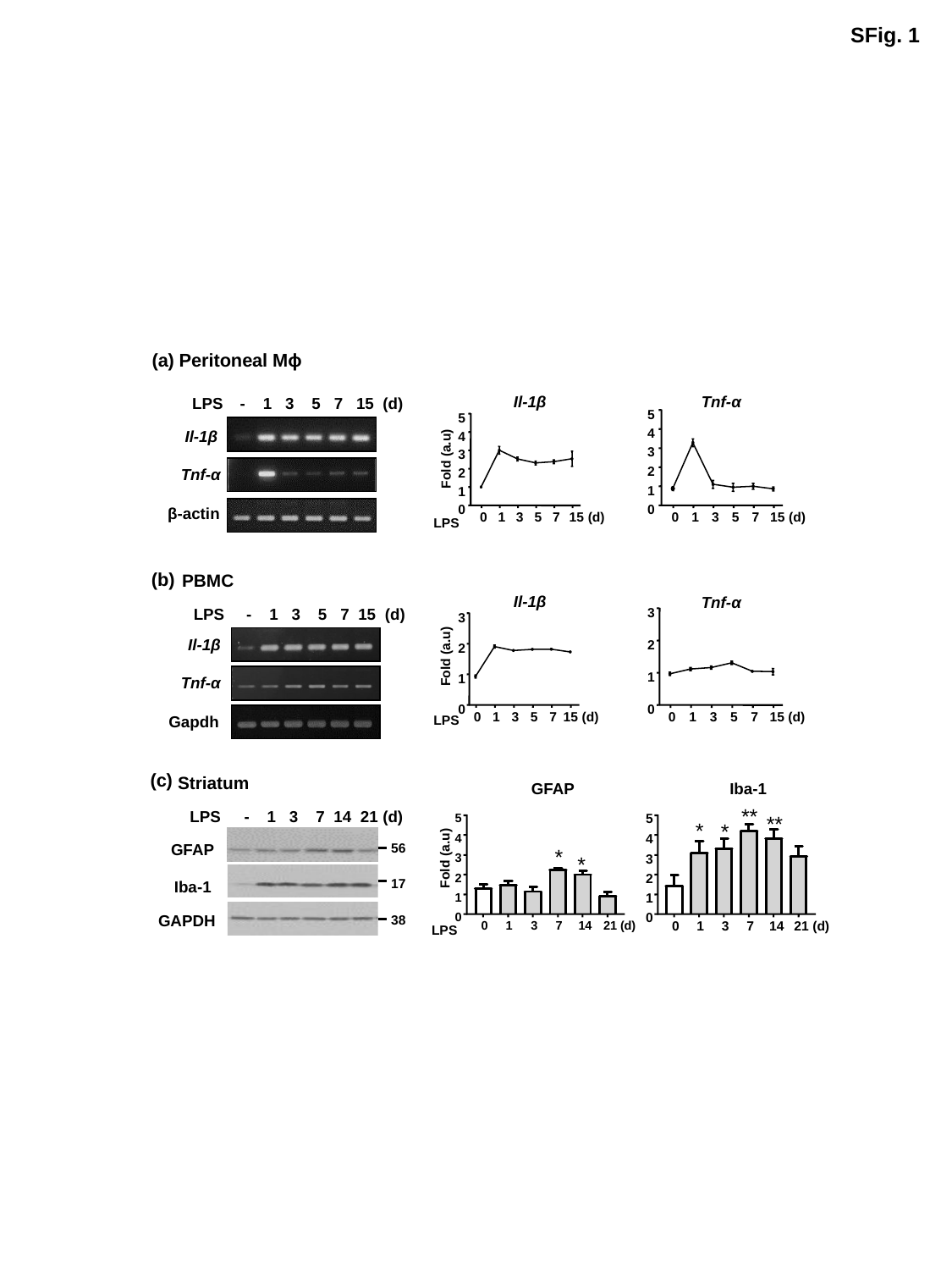

SFig. 1
(a)
Peritoneal Mϕ
LPS
- 1 3 5 7 15 (d)
Il-1β
5
4
Fold (a.u)
3
2
1
0
LPS
1
3
5
7
15 (d)
0
Tnf-α
5
4
3
2
1
0
0
1
3
5
7
15 (d)
Il-1β
Tnf-α
β-actin
(b)
PBMC
Il-1β
3
2
Fold (a.u)
1
0
LPS
0
1
3
5
7
15 (d)
Tnf-α
3
2
1
0
0
1
3
5
7
15 (d)
LPS
- 1 3 5 7 15 (d)
Il-1β
Tnf-α
Gapdh
(c)
Striatum
LPS
- 1 3 7 14 21 (d)
GFAP
56
17
Iba-1
GAPDH
38
GFAP
5
4
3
2
1
0
0
1
3
7
14
21 (d)
*
Fold (a.u)
*
Iba-1
**
**
5
*
*
4
3
2
1
0
0
1
3
7
14
21 (d)
LPS

## Slide 2
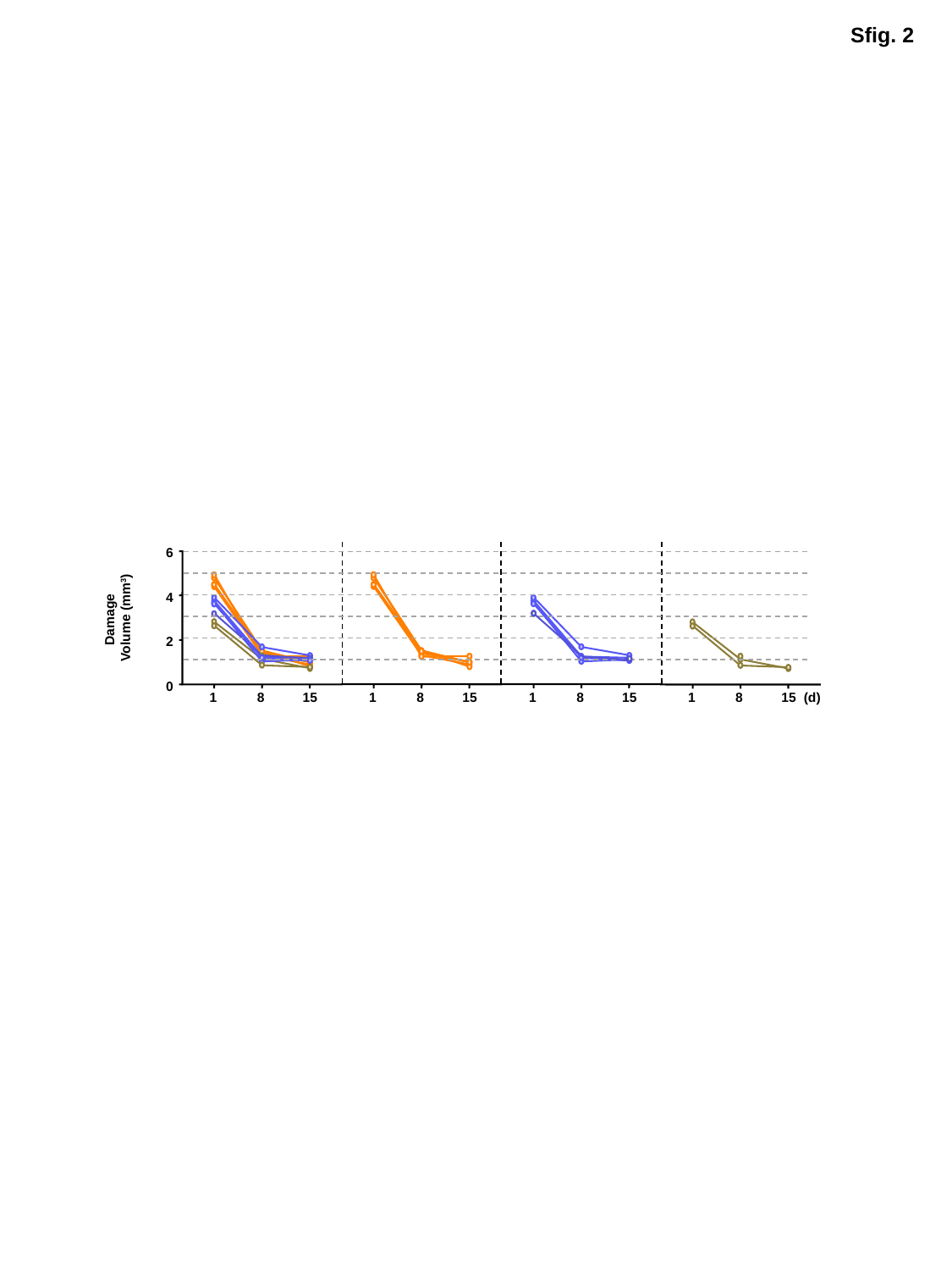

Sfig. 2
Damage
Volume (mm3)
6
4
2
0
1
8
15
1
8
15
1
8
15
1
8
15 (d)

## Slide 3
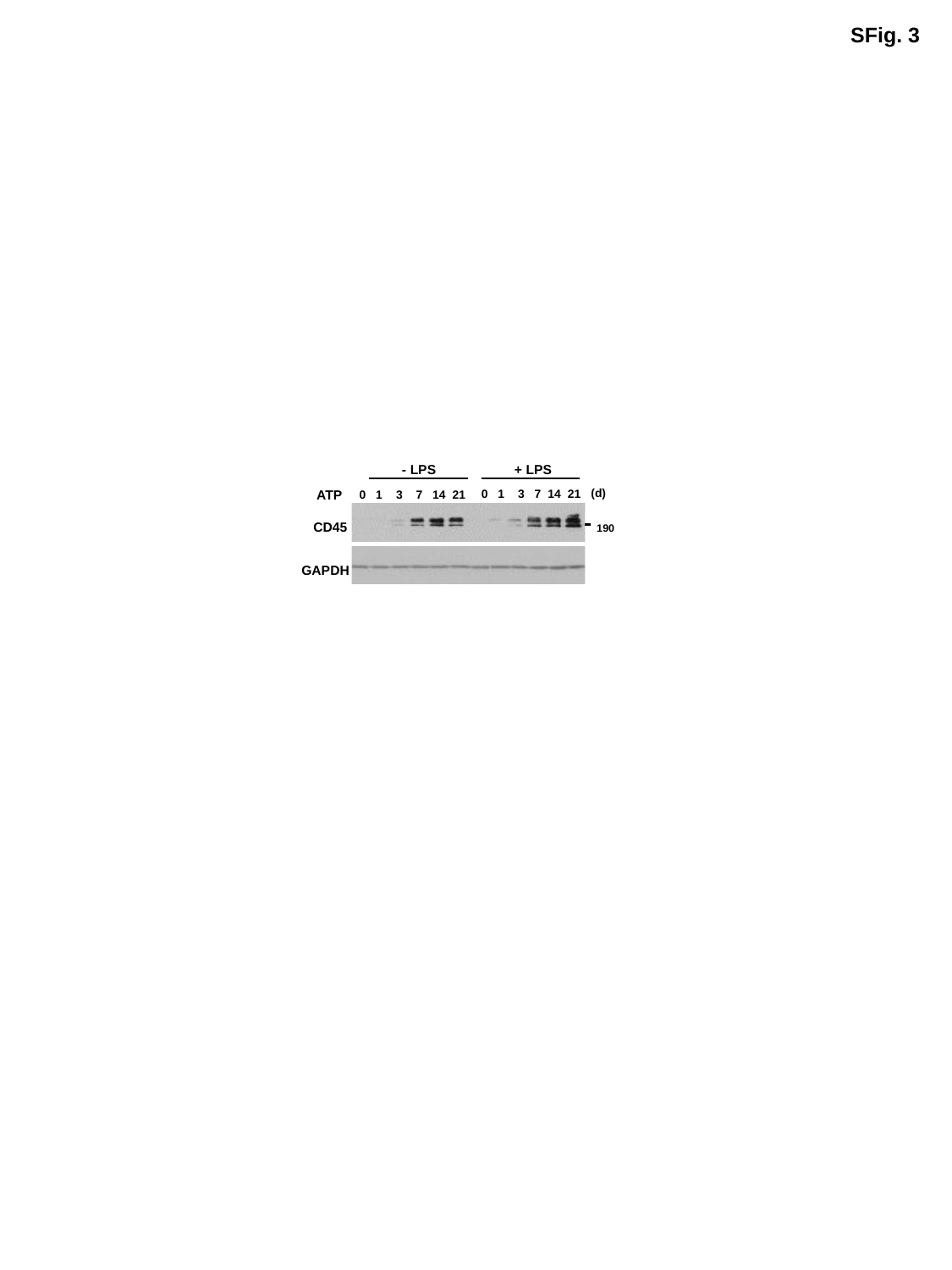

SFig. 3
- LPS
+ LPS
(d)
0 1 3 7 14 21
ATP
 0 1 3 7 14 21
190
CD45
GAPDH

## Slide 4
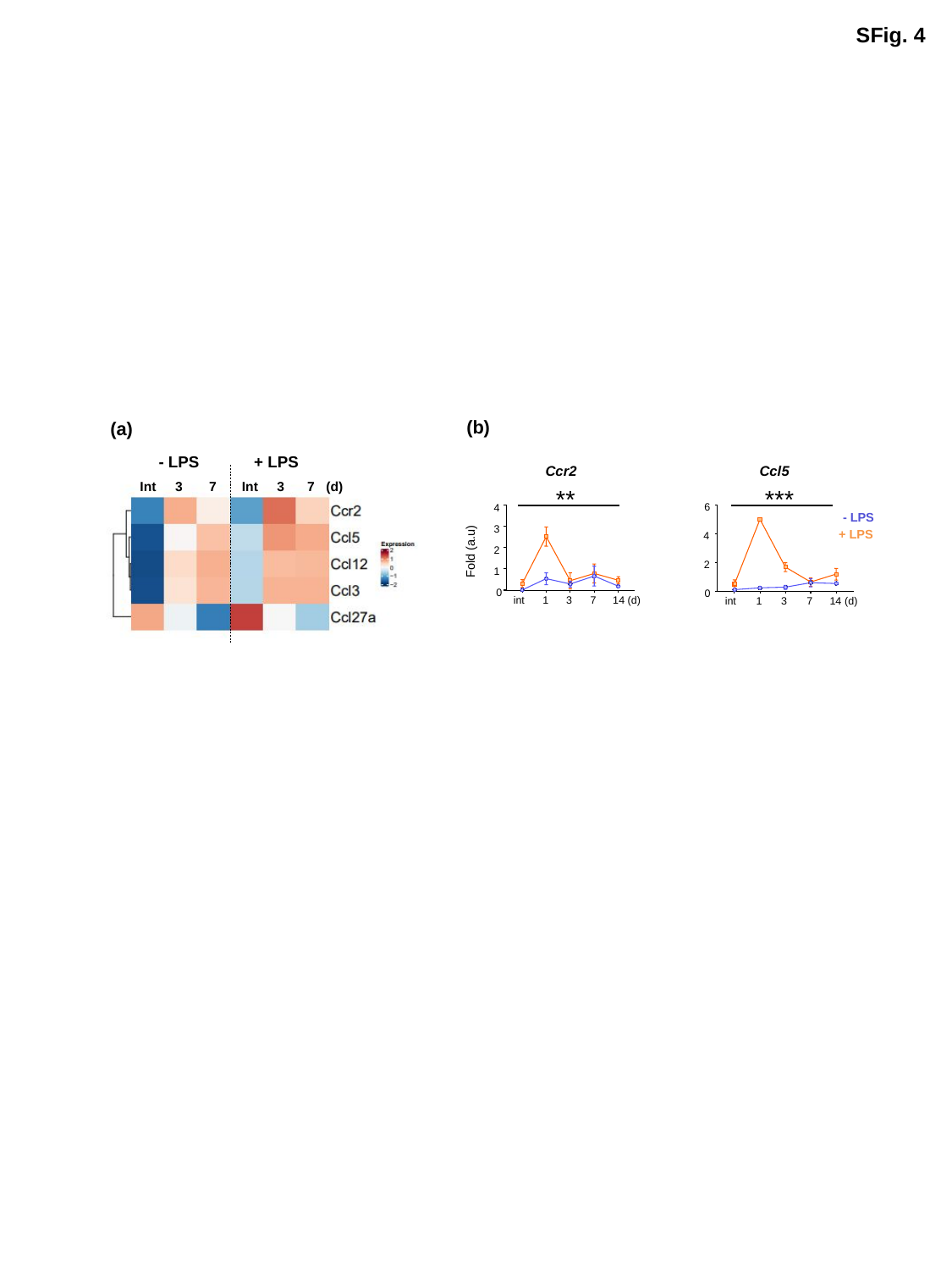

SFig. 4
(b)
(a)
- LPS
+ LPS
Int 3 7 (d)
Int 3 7
 Ccr2
**
4
3
Fold (a.u)
2
1
0
int
1
3
7
14 (d)
 Ccl5
***
6
4
2
0
int
1
3
7
14 (d)
 - LPS
 + LPS

## Slide 5
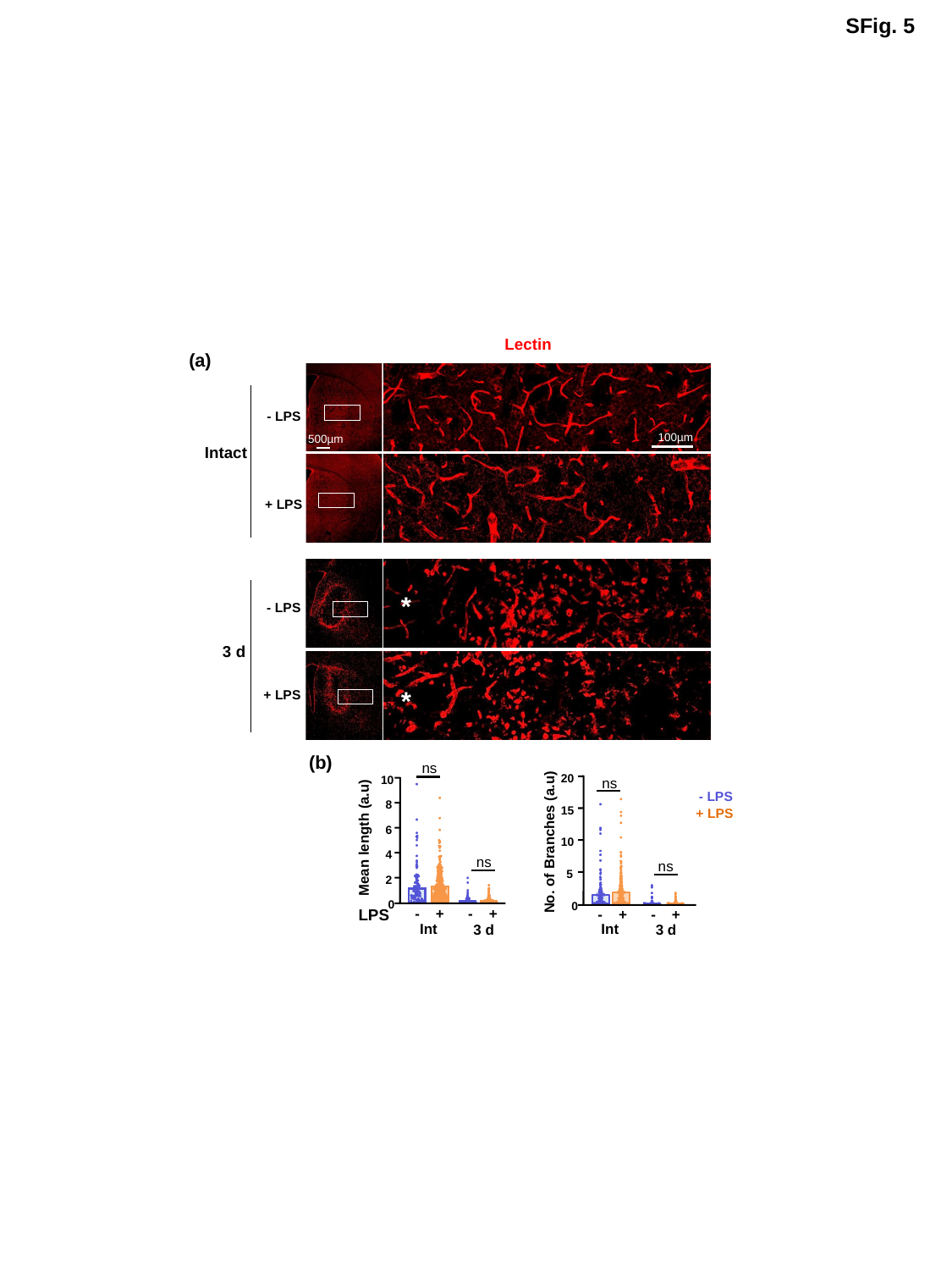

SFig. 5
Lectin
(a)
100µm
- LPS
500µm
Intact
+ LPS
*
- LPS
3 d
*
+ LPS
(b)
ns
ns
20
10
- LPS
+ LPS
8
15
6
 Mean length (a.u)
 No. of Branches (a.u)
10
ns
4
ns
5
2
0
- + - +
0
LPS
- + - +
Int
Int
3 d
3 d

## Slide 6
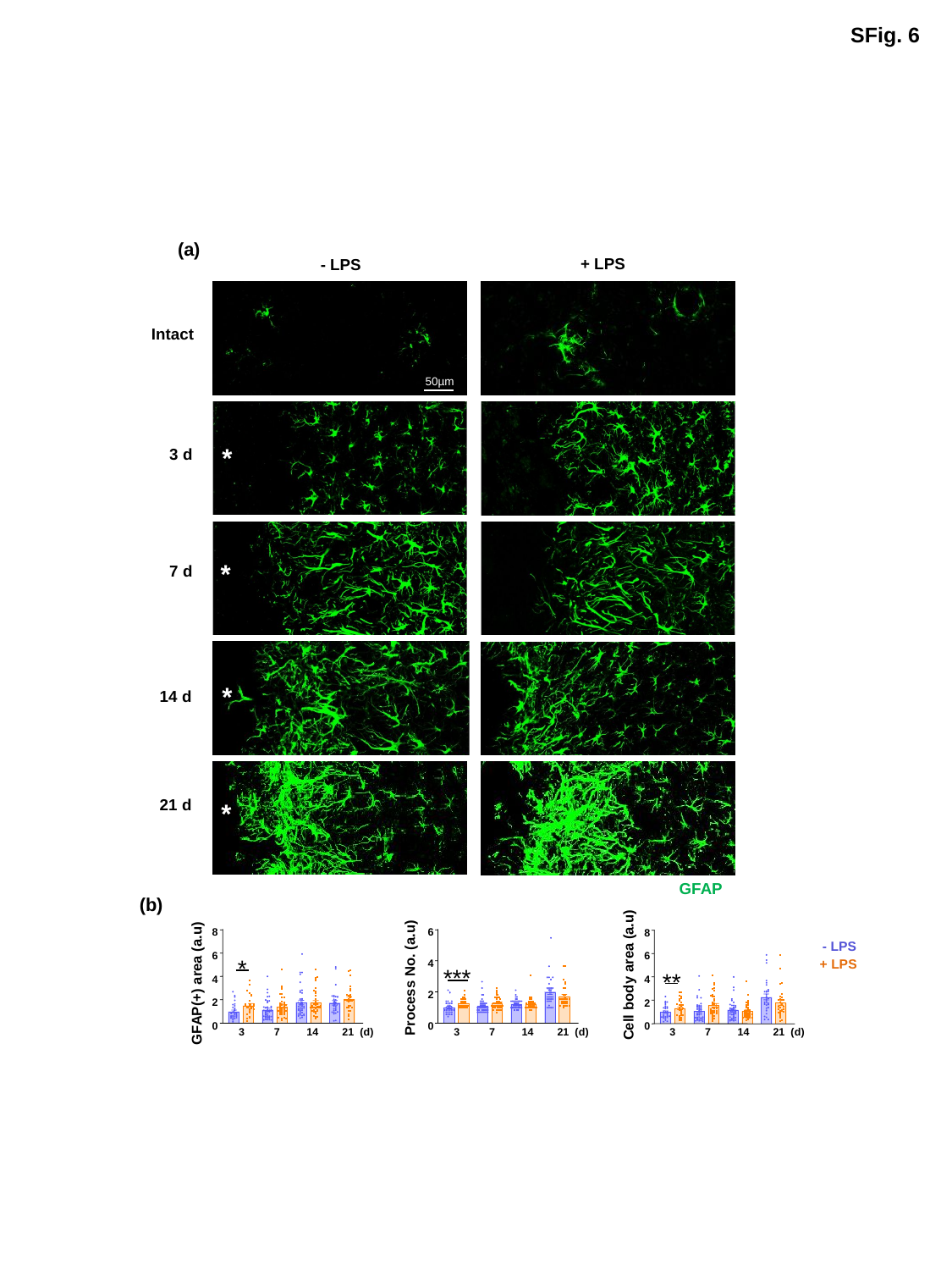

SFig. 6
(a)
+ LPS
- LPS
50µm
Intact
*
3 d
*
*
7 d
*
14 d
21 d
*
GFAP
(b)
8
6
**
4
2
0
Cell body area (a.u)
3 7 14 21 (d)
6
***
4
Process No. (a.u)
2
0
8
*
6
GFAP(+) area (a.u)
4
2
0
3 7 14 21 (d)
- LPS
+ LPS
3 7 14 21 (d)
